# The Impact of Financial Interest in Intensity-Modulated Radiation Therapy on the Utilization of Radiation Therapy for Treatment of Newly Diagnosed Prostate Cancer: A Single Center Experience

**DOI:** 10.5402/2012/759258

**Published:** 2012-03-11

**Authors:** Xiaolong S. Liu, Joseph C. Zola, David E. McGinnis, Mehrdad Soroush, Leigh G. Bergmann, David J. Ellis, James F. Squadrito, Ilia S. Zeltser

**Affiliations:** The Bryn Mawr Urology Group, Academic Urology, Bryn Mawr, PA 19010, USA

## Abstract

*Objective*. As recent participants in an integrated prostate cancer (PCa) care center, we sought to evaluate whether financial investment in an intensity-modulated radiation therapy (IMRT) center resulted in an increased utilization of radiation therapy in our patients with newly diagnosed PCa. *Materials & Methods*. Following institutional review board approval, we retrospectively reviewed the records of all consecutive patients who were diagnosed with prostate cancer in the 12 months prior to and after investment in IMRT. Primary treatment modalities included active surveillance (AS), brachytherapy (BT), radiation therapy (XRT), radical prostatectomy (RP), and androgen deprivation therapy (ADT). Treatment data were available for all patients and were compared between the two groups. *Results*. A total of 344 patients with newly diagnosed PCa were evaluated over the designated time period. The pre-investment group totaled 198 patients, while 146 patients constituted the post-investment group. Among all patients evaluated, there was a similar rate in the use of XRT (20.71% versus 20.55%, *P* = 1.000) pre- and post-investment in IMRT. *Conclusions*. Financial interest in IMRT by urologists does not impact overall utilization rates among patients with newly diagnosed PCa at our center.

## 1. Introduction

Prostate cancer (PCa) is the most common solid organ malignancy in American men with global statistics mirroring those found in the United States [1]. Screening with prostate specific antigen (PSA) has resulted in a significant stage migration such that the majority of new cases of PCa are now detected while the disease is still clinically localized [2]. These patients can choose from several treatment options and must weigh the potential morbidity of each treatment modality on their quality of life. In the vast majority of cases, urologists are the primary physicians that diagnose these patients with PCa and are typically involved in the initial work up, discussion of all treatment options, and counseling of patients. Urologists are not only intimately involved with the treatment of the primary disease but also the consequential treatment-related complications while overseeing the long-term followup of these patients.

Several centers have been recently established where urologists partner with radiation oncologists acquire ownership interest in intensity-modulated radiation therapy (IMRT) equipment and provide integrated prostate cancer care. Although not yet validated in the literature, this may allow for improved quality of care and decreased cost. Unfortunately, these efforts have been much maligned in both the media and radiation oncology literature as conduits to increased revenue for the urologists with only debatable patient benefit [3, 4]. These reports have not yet been supported by data.

After acquiring financial interest in an integrated prostate cancer center, we sought to evaluate whether our investment in IMRT resulted in an increased utilization of radiation therapy in our patients with newly diagnosed prostate cancer.

## 2. Materials and Methods

 In September of 2008, we acquired financial interest in an integrated PCa center offering IMRT. Following institutional board approval, we identified all patients who were diagnosed with PCa in the 12 months before and after the center became operational. Newly diagnosed cases of PCa were identified by searching our electronic medical record using both prostate biopsy CPT codes (transrectal ultrasound-guided [TRUS] needle biopsy of the prostate, 55700/76942/76872) and the ICD-9 prostate cancer diagnostic code (prostate cancer, 185.0). All men were diagnosed with PCa after pathologic review of biopsy needle cores obtained after TRUS. Indications for biopsy included elevated PSA, abnormal digital rectal exam, abnormal PCA 3 test, and/or strong family history of PCa. Prostate biopsies were performed utilizing a routine sextant pattern with at least 12 cores obtained. In cases where clinically indicated, an extended template biopsy with a higher number of cores obtained was utilized. The medical records of these patients were retrospectively reviewed and the data pertaining to the patients' demographics, cancer parameters, and initial PCa treatment modality were extracted.

Patients were assigned to two discrete groups based on the date of their PCa diagnosis as it related to the date of the first availability of IMRT at our integrated prostate center. All consecutive patients diagnosed with PCa on a biopsy within 12 months prior to availability of IMRT constituted the pre-investment group and all consecutive patients diagnosed with prostate cancer on a biopsy in the 12 months following initiation of IMRT services constituted the post-investment group. No patient, regardless of comorbidity, was excluded from the analysis. The primary treatments received were designated as active surveillance (AS), brachytherapy (BT), radiation therapy (XRT), radical prostatectomy (RP), and androgen deprivation therapy (ADT). Treatment data were available for all patients and were stratified by the patient's age and Gleason score. The age of 70 years old served as a cut-off point as in our clinical practice most of these patients are deemed suboptimal surgical candidates because of increased risk of postoperative urinary incontinence and erectile dysfunction.

Our integrated PCa center was established in collaboration with an academic radiation oncology department from a National Cancer Institute (NCI) designated cancer center with a nationwide reputation for clinical and academic excellence. The radiation oncologists that treat our patients are not employed by the cancer center, but serve as full time academic faculty and have no financial interest in IMRT. Radiation oncology residents have the opportunity to participate in all aspects of planning and delivery of radiation therapy. The center also employs a nurse-practitioner charged exclusively with the coordination and support of on-going clinical research programs.

Patients who were referred to us for second opinion with biopsy-proven PCa as well as men referred to us specifically for robotic-assisted laparoscopic radical prostatectomy were excluded to eliminate the potential biases resulting from the treatment recommendations rendered by an outside urologist. The overwhelming majority of these patients choose surgical therapy and their inclusion would skew the results toward lower utilization rates of XRT. Statistical analysis was performed using GraphPad InStat version 3.0, La Jolla, CA. Unpaired *t*-tests, Chi-squared tests, and Fisher's exact tests were implemented as appropriate. *P* value < 0.05 was considered to represent statistical significance.

## 3. Results

**Figure 1:**
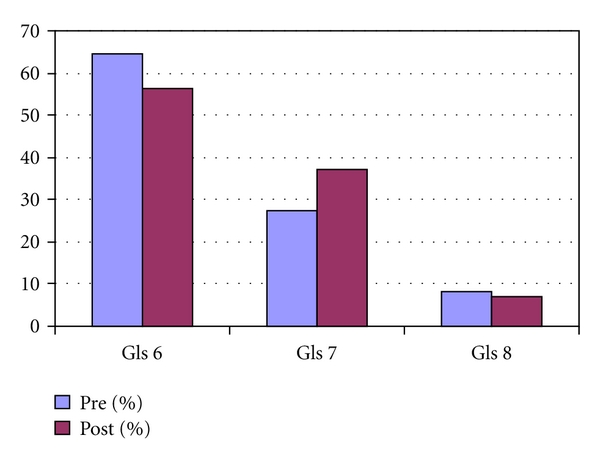
Gleason score distribution.

 A total of 344 patients were diagnosed with PCa on TRUS biopsy over the designated 24-month time period. Of the total patient population, 198 men were diagnosed with PCa in the 12 months preceding availability of IMRT, while 146 men constituted the post-investment group. Patient and cancer characteristics were similar between the two groups (Table 1 and Figure 1). The percentage of patients with Gleason 7 PCa was higher in the post-investment group but did not reach statistical significance, *P* = 0.073 (Figure 1).

**Figure 2:**
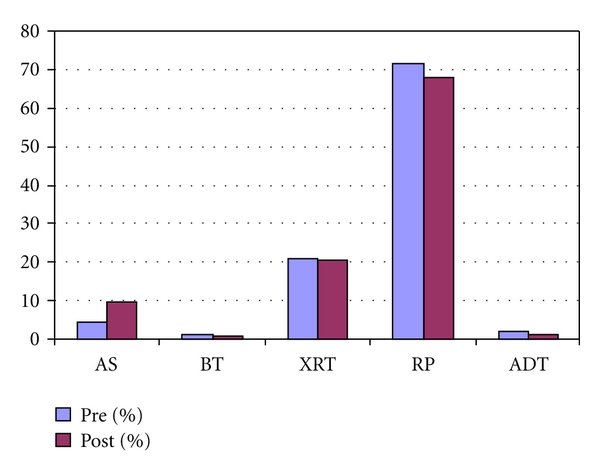
Overall treatment distribution.

Overall, the use of radiation therapy for those patients with newly diagnosed PCa following investment in IMRT (20.55% versus 20.71%, *P* = 1.00) was similar between the two patient populations (Table 2 and Figure 2). The number of patients treated with RP (67.81% versus 71.72%, *P* = 0.729), AS (9.59% versus 4.55%, *P* = 0.177), ADT (1.37% versus 2.02%, *P* = 0.999), and BT (0.68% versus 1.01%, *P* = 0.999) were not significantly different between post- and pre-investment groups.

**Figure 3:**
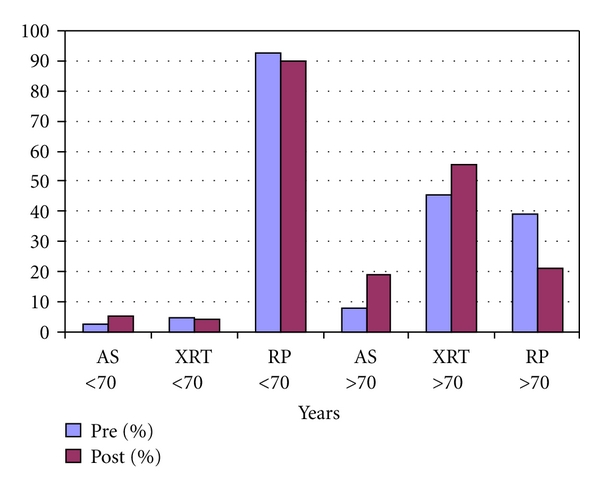
Treatment choice stratified by patient age.

While overall treatment trends afford succinct analysis, clinical decisions regarding treatment of PCa are often driven by a multitude of patient-specific factors. As such, the data was analyzed stratifying both Gleason score and age (70 years old serving as a cutoff point). Treatments stratified by patient age are shown in Table 3 and Figure 3. Despite the increased incidence of Gleason score ≥7 disease in the post-investment group, there was no significant difference between the groups in all treatment patterns in men less than 70 years of age (Table 3). An increase was found in the use of XRT in men older than 70 years of age in the pre-investment group (45.45%) as compared to men following acquisition of IMRT (55.32%), but this did not reach statistical significance (*P* = 0.355).

**Figure 4:**
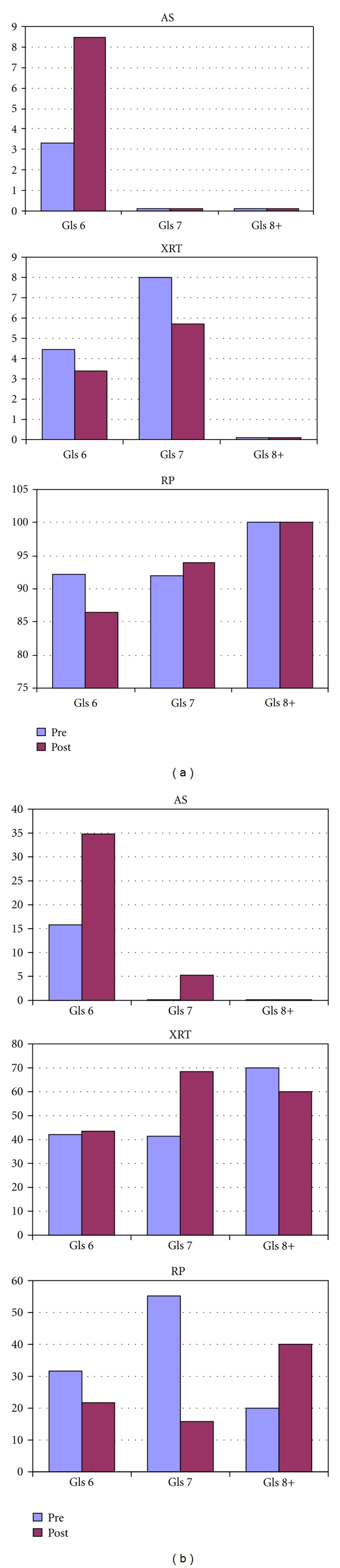
(a) Treatment choice stratified by gleason score and age (<70 yrs). (b) Treatment choice stratified by gleason score and age (>70 yrs).

Analyzed by age and Gleason score simultaneously, there was no difference between the treatment groups in patients younger than 70 regardless of the Gleason score, Table 4(a) and Figure 4(a). For men 70 years or older with Gleason 6 disease, there was a trend toward increased use of AS (34.78% versus 15.79%) and decreased use of RP (21.74% versus 31.58%), but did not reach statistical significance (Table 4(b)). There was no difference (43.48% versus 42.11%) in the use of XRT for men over 70 with Gleason 6 disease. For patients with Gleason 7 disease, there was a statistically significant increase in the utilization of XRT (pre-investment 41.38% versus post-investment 68.42%, *P* = 0.035) and decrease in the use of RP in the post-investment group (15.79% versus 55.17%, *P* = 0.006) seen in Table 4(b) and Figure 4(b). There was no difference between treatment groups in men over 70 with Gleason 8 disease. 

## 4. Discussion

 External beam radiation therapy is widely used and is an effective treatment option for localized PCa. There is now Level I evidence that high-dose radiation therapy decreases the risk of biochemical failure in men with clinically localized prostate cancer as compared to conventional dose conformal radiation [5–7]. This improvement, however, comes at a cost of increased gastrointestinal (GI) and genitourinary (GU) toxicity. Zietman et al. observed that 2% of men receiving high-dose radiation experienced acute urinary or rectal morbidity of radiation therapy oncology group (RTOG) grade 3 or greater [7]. Furthermore, about 3% of patients experienced late RTOG grade 3 morbidity.

Intensity modulated radiation therapy allows for delivery of radiation with greater conformality to the target volume compared with traditional 3 D technique. Several randomized trials have shown that IMRT reduced GI and GU toxicity compared with 3 D conformal radiation [8–10]. Zelefsky et al. were able to deliver 81 Gy with less than 2% of grade 2 rectal morbidity and no grade 4 or greater rectal complications in patients with clinically localized PCa. Furthermore, IMRT has been shown to reduce the acute and late GI toxicity of patients treated with high-dose radiation therapy and adjuvant androgen deprivation as compared to 3 D conformal radiotherapy [8]. This reduction in GU and GI morbidity has made IMRT extremely popular in the delivery of high-dose external beam radiation for patients with clinically localized PCa in the United States.

Since 2004 several large urology groups in partnership with radiation and medical oncologists have established centers of integrated prostate cancer care. These centers provide for a collaborative approach to the treatment of PCa. The integrated care model is patient-centered and disease specific, where the equipment and the staff are dedicated to the treatment of PCa and no other disease entity. Although yet to be validated, this model may potentially result in better recognition and management of treatment-related complications, improved access to care, and increased experience with each treatment modality and thus better clinical outcomes.

Recently, these centers have become the targets of intense criticism [3, 4]. The detractor's claim that integrated PCa care centers lead to self-referral by financially motivated urologists and radiation oncologists and result in over-utilization of IMRT contributing to the increased cost of health care. They further claim that these centers have a negative impact on residency training in radiation oncology by shifting patients away from the academic radiation oncology training programs. Unfortunately, these claims are not substantiated by data, but rather rely on indirect analysis of Medicare claims and a 12% negative impact report from a single 3-point questionnaire survey of 81 radiation oncology training programs [3, 4].

To our knowledge, this is a first study conducted to directly evaluate whether financial interest in IMRT as part of the integrated prostate cancer care model changed treatment recommendations for newly diagnosed patients with prostate cancer. We compared the distribution of treatments choices of all consecutive patients diagnosed with prostate cancer on a biopsy in our practice during a 12-month period prior to acquiring financial interest in IMRT to a 12-month period following that acquisition. Our analysis revealed that overall there was a small, but statistically insignificant decrease in the use of radiation therapy and radical prostatectomy and a small increase in the use of active surveillance following investment in IMRT. The increased use of active surveillance is likely due to the emergence of data from several large trials supporting the safety and efficacy of this approach in appropriately selected patients [11–13].

Once the data were stratified by Gleason score and patient age, a statistically significant increase in the use of radiation was found in men over 70 with Gleason 7 disease (41.3% versus 68.3%). However, because of the overall low number of patients in this subgroup, this increase was due to a single patient difference between the groups (12 versus 13).

These findings are not surprising as we believe that several important attributes of our integrated prostate cancer program provide for many patient benefits without the recently theorized, yet unsubstantiated risks of overutilization of IMRT. As previously described, our center was established in collaboration with an academic radiation oncology department from an NCI designated cancer center with a nationwide reputation for clinical and academic excellence. The radiation oncologists that treat our patients are not employed by the integrated prostate cancer center, but rather serve as full-time academic faculty and have no financial interest in IMRT. Furthermore, the final determination on whether a patient is an appropriate candidate for primary or adjuvant radiation therapy is made entirely by the treating radiation oncologist. Radiation oncology residents have the opportunity to participate in all aspects of planning and delivery of IMRT, thus deriving an educational benefit from this partnership. All patients are considered and frequently enrolled into the RTOG-sponsored randomized trials. The center employs a nurse-practitioner charged exclusively with coordination and support of the clinical research program. Additionally, we are privileged to have CME accreditation by our state medical society and conduct regularly scheduled multidisciplinary morbidity and mortality conferences and discussions of challenging cases. Finally, we maintain a very high volume surgical program that ranks second in the number of radical prostatectomies performed annually in the greater Philadelphia, PA, USA.

Our study has several limitations that must be acknowledged. First, this study is underpowered due to a fairly low number of patients and therefore the results of our statistical analysis must withstand the test of a larger trial for our conclusions to be validated. Second, even though we attempted to minimize limitations of the retrospective study design by including all consecutive patients diagnosed with prostate cancer within the 24-month period, the selection bias inherent in retrospective study design was not completely eliminated. Third, we did not include patients undergoing adjuvant or salvage radiation therapy in the trial and therefore did not ascertain the effect of financial interest in IMRT on utilization of radiation in these patients. Finally, our findings may not be applicable to other integrated prostate cancer centers because of the unique structure of our specific program.

## 5. Conclusion

Financial interest in IMRT does not result in an increased utilization of radiation therapy in the treatment of newly diagnosed patients with clinically localized prostate cancer in our integrated prostate cancer center. A large prospective trial is warranted to validate these initial findings.

## Figures and Tables

**Table 1:** Patient Characteristics.

	Pre-investment	% Pre-investment	Post-investment	% Post-investment	*P* value
Age (years)	67.5		66.1		0.174
PSA (ng/mL)	5.56		6.54		0.291
Gls 6	128	64.65	82	56.16	0.073
Gls 7	54	27.27	54	36.99	0.073
Gls 8+	16	8.08	10	6.85	0.293

**Table 2:** Overall Treatment Distribution.

	Pre-investment	% Pre-investment	Post-investment	% Post-investment	*P* value
AS	9	4.55	14	9.59	0.177
BT	2	1.01	1	0.68	0.999
XRT	41	20.71	30	20.55	1.000
RP	142	71.72	99	67.81	0.729
ADT	4	2.02	2	1.37	0.999

**Table 3:** Treatment Choice Stratified by Age.

	Pre-investment				Post-investment			
	<70 yrs	%	>70 yrs	%	<70 yrs	%	>70 yrs	%
Gls 6	90	74.38	38	49.35	59	59.60	23	48.94
Gls 7	25	20.66	29	37.66	35	35.35	19	40.43
Gls 8+	6	4.96	10	12.99	5	5.05	5	10.64
AS	3	2.48	6	7.79	5	5.05	9	19.15
BT	0	0.00	2	2.60	1	1.01	0	0.00
XRT	6	4.96	35	45.45	4	4.04	26	55.32
RP	112	92.56	30	38.96	89	89.90	10	21.28
ADT	0	0.00	4	5.19	0	0.00	2	4.26

**Table:** (a)

	Pre-investment		Post-investment	
	<70 yrs	%	<70 yrs	%
*Gls 6*				
AS	3	3.33	5	8.47
XRT	4	4.44	2	3.39
RP	83	92.22	51	86.44

*Gls 7*				
AS	0	0	0	0
XRT	2	8	2	5.71
RP	23	92	33	94.29

*Gls 8+*				
AS	0	0	0	0
XRT	0	0	0	0
RP	6	100	5	100

**Table:** (b)

	Pre-Investment		Post-Investment	
	>70 y	%	>70 y	%
*Gls 6*				
AS	6	15.79	8	34.78
XRT	16	42.11	10	43.48
RP	12	31.58	5	21.74

*Gls 7*				
AS	0	0	1	5.26
XRT	12	41.38	13	68.42
RP	16	55.17	3	15.79

*Gls 8+*				
AS	0	0	0	0
XRT	7	70	3	60
RP	2	20	2	40
